# Identification and Profiling of microRNAs in Goat Endometrium during Embryo Implantation

**DOI:** 10.1371/journal.pone.0122202

**Published:** 2015-04-17

**Authors:** Yuxuan Song, Xiaopeng An, Lei Zhang, Mingzhe Fu, Jiayin Peng, Peng Han, Jingxing Hou, Zhanqin Zhou, Bingyun Cao

**Affiliations:** College of Animal Science and Technology, Northwest A&F University, Yangling, Shaanxi, 712100, P.R. China; Kunming University of Science and Technology, CHINA

## Abstract

**Background:**

MicroRNAs (miRNAs) are short, highly conserved small noncoding RNAs that had fundamental roles in post-transcriptional gene expression, and they are crucial for proper control of biological processes and known to participate in embryo implantation. However, miRNA expression profiles in the pre-receptive and receptive phases of the goat endometrium during embryo implantation are unknown.

**Results:**

A total of 1,069 and 847 miRNAs were expressed in receptive (R) and pre-receptive (P) goat endometrium, and 632 miRNAs were co-expressed in both phases. We identified 545 (50.98%) known miRNAs in the R library and 522 (61.63%) in the P library. There were 110 up-expressed miRNAs and 33 down-expressed miRNAs in receptive endometrium compared with the pre-receptive endometrium meeting the criteria of *P*-values< 0.05. Moreover, GO and KEGG analysis of the target genes of the differentially expressed miRNAs revealed some candidate miRNAs, genes and pathways that may involve in the formation of the receptive endometrium. Based on stem-loop RT-qPCR, 15 miRNAs were detected and the results suggested that the majority of the miRNA expression data measured by Solexa deep sequencing could represent actual miRNA expression levels.

**Conclusions:**

Our data revealed the first miRNA profile related to the biology of the goat receptive endometrium during embryo implantation, and the results suggested that a subset of miRNAs might play important roles in the formation of endometrial receptivity. Thus, elucidating the physiological roles of endometrial miRNAs will help us better understand the genetic control of embryo implantation in goats.

## Introduction

Embryo implantation is a crucial step in mammalian reproduction [1]. Endometrium sensitivity to embryo implantation is classified as a pre-receptive phase and a receptive phase in mammals [2, 3]. The development of a receptive endometrium is a complex process and appropriate endometrial preparation with stromal proliferation and epithelial differentiation is stimulated prior to embryo–uterine interactions [4], which is a spatial and temporal phenomenon known as the ‘window of implantation’ [5, 6]. During this restricted period, a folded endometrial epithelial bilayer develops and the endometrium acquires the adhesive properties that allow embryo adhesion and its subsequent invasion [7].

Some factors act directly on the endometrium, and regulated endometrial functions promote the interactions between endometrium and embryo [8]. Accumulated evidence suggests that hormones [9], homeobox proteins [10], morphogens [11] and other factors participate in the preparation of endometrial receptivity [12]. Moreover, molecular studies have extensively investigated the expression and regulation of different factors connected with endometrial receptivity, with the list of possible genes involved in the establishment of a receptive endometrium increasing exponentially in rats, pigs, and humans [13, 14]. Gaining a solid understanding of the molecular mechanisms underlying endometrium remodeling is important for investigation into reproduction of female goats, which is the most economically important component in commercial goat breeding [15, 16].

Endometrial receptivity is a dynamic process that was believed to be under post-transcriptional regulation because several genes expressed in the endometrium were identified as being epigenetically regulated [17]. MicroRNAs (miRNAs), with an average length between 18 nt and 26 nt [18], are a new class of small non-coding RNAs that function as negative regulators by repressing gene expression, inhibiting translation or regulating mRNA degradation [19]. miRNA recognition is based on either incomplete or complete complementarity to target sequences within the 3’ untranslated region (3’UTR) at the post-transcriptional level [7, 20]. Between 1 and 5% of genes are predicted to encode miRNA, and these miRNA may regulate the expression of as many as 30% of mRNA [21, 22]. Mature miRNA are synthesized through a multi-step process that concludes with the cleavage of the pre-miRNA stem-loop by the RNase III enzyme Dicer1. A single miRNA can target hundreds of mRNAs [23]. Thus, miRNA are potential regulators of endometrial receptivity at the post-transcriptional level via targeting of mRNAs for degradation or translational repression [19, 20]. Differentially expressed miRNAs have been identified in the mouse, rat and human uterus [14, 24], as well as the porcine placenta [25, 26].

Nevertheless, little information was available on miRNA expressed in the goat endometrium, and differences between the pre-receptive and receptive phases during embryo implantation were unknown. Focusing on these points, we aimed to gain more understanding of the complex regulation of endometrial receptivity in healthy multiparous dairy goats by analyzing miRNA expression in the endometrium at gestational day 5 (pre-receptive endometrium phase) and gestational day 15 (receptive endometrium phase) using Illumina Solexa technology. And then we investigated the known miRNAs, potential novel miRNAs, differentially expressed miRNAs and their target genes. Given the biological functions of miRNAs at the posttranscriptional level and genes usually interact with each other to play their roles, GO enrichment and KEGG pathway analysis were also made in this study. The results would provide further understanding of the miRNAs role in the regulation mechanism of the endometrial receptivity.

## Results

### Overview of sequencing data

**Fig 1:**
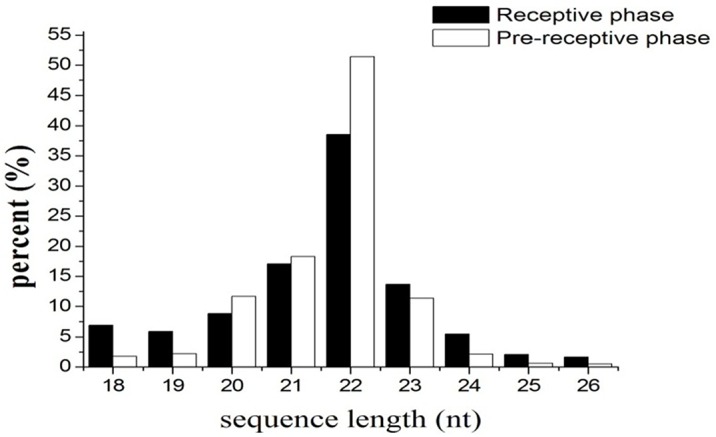
The size distribution of the small RNAs in R and P libraries.

To systematically identify small RNAs and changes in the expression level of miRNAs between the pre-receptive and receptive phases of the endometrium in Xinong Saanen dairy goats, we purified and sequenced small RNAs from the goat endometrium. A total of 7,717,603 and 6,307,668 raw reads were obtained from the R (receptive) and P (pre-receptive) endometrium, respectively. To assess the efficiency of Solexa sequencing and the quality of the sequences, all reads were annotated and classified by aligning against the miRBase 20.0 database (ftp://mirbase.org/pub/mirbase/CURRENT/), the Rfam database (http://rfam.janelia.org), the Repbase database (http://www.girinst.org/repbase), the Genome database (http://goat.kiz.ac.cn/GGD/download.htm) and the mRNA database (http://goat.kiz.ac.cn/GGD/download.htm). Small RNAs were classified into different categories according to their annotations. Next, 3’ ADT and length filter, junk reads, Rfam, mRNA, repeats, rRNA, tRNA, snRNA and snoRNA sequences, as well as other Rfam RNA sequences, were separated out and discarded. As a result, 4,233,874 clean reads representing 284,622 unique sequences from the R library were obtained, and 5,386,427 clean reads representing 139,040 unique sequences from the P library (Table 1). The lengths of the majority of the clean reads ranged between 18 and 26 nt. The most abundant class size in the small RNA sequence distribution was 22 nt, which accounted for 38.54% and 51.43% of reads in the R and P libraries, respectively, followed by 21 nt (17.03% and 18.27%), 23 nt (13.65% and 11.33%) and 20 nt (8.81% and 11.68%), suggesting that they were typical small RNA (Fig 1, Table 2).

**Table 1:** Summary of reads from raw data to cleaned sequences for small RNAs in Receptive Versus Pre-receptive Endometrium in goat.

Item	R library (receptive endometrium)		P library (pre-receptive endometrium)	
	Total (%)	Unique (%)	Total (%)	Unique (%)
Raw reads	7717603(100%)	1028383(100%)	6307668(100%)	401202(100%)
3’ADT&length	2461440(31.89%)	618853(60.18%)	589698(9.35%)	215293(53.66%)
Junk reads	6783(0.09%)	3180(0.31%)	1778(0.03%)	1188(0.3%)
Rfam	801419(10.38%)	41322(4.02%)	274894(4.36%)	21723(5.41%)
mRNA	236734(3.07%)	80731(7.85%)	62332(0.99%)	24153(6.02%)
Repeats	104302(1.35%)	12589(1.22%)	39894(0.63%)	5197(1.3%)
rRNA	601798(7.8%)	17270(0.22%)	191856(3.04%)	9341(0.15%)
tRNA	96971(1.26%)	6847(0.09%)	38937(0.62%)	2487(0.04%)
snoRNA	23920(0.31%)	6185(0.08%)	17307(0.27%)	4362(0.07%)
snRNA	20979(0.27%)	4648(0.06%)	7101(0.11%)	2284(0.04%)
other Rfam RNA	57751(0.75%)	6372(0.08%)	19693(0.31%)	3249(0.05%)
Clean reads	4233874(54.86%)	284622(%)	5386427(85.39%)	139040(34.66%)

Notes: 3’ADT & length filter: reads removed due to 3ADT not found and length with<18 and >26 were remove (for animals). Junk: > = 2N, > = 7A, > = 8C, > = 6G, > = 7T, > = 10 Dimer, > = 6 Trimer, or > = 5 Tetramer. Rfam: Collection of many common non-coding RNA families except micro RNA; http://rfam.janelia.org. mRNA: http://goat.kiz.ac.cn/GGD/download.htm; Repeats: Prototypic sequences representing repetitive DNA from different eukaryotic species; http://www.girinst.org/repbase, there is overlap in mapping of reads with rRNA, tRNA, snRNA, snoRNA and repeats.

**Table 2:** Length distribution of sRNAs in this study.

Length	R library		P library	
	Number	Percentage (%)	Number	Percentage (%)
18	290680	6.87	94892	1.76
19	249942	5.9	118829	2.21
20	373005	8.81	629143	11.68
21	721054	17.03	984344	18.27
22	1631903	38.54	2770035	51.43
23	577842	13.65	610482	11.33
24	230879	5.45	116718	2.17
25	88735	2.1	33927	0.63
26	69834	1.65	28057	0.52
Total cleaned Reads	4233874	100	5386427	100

**Fig 2:**
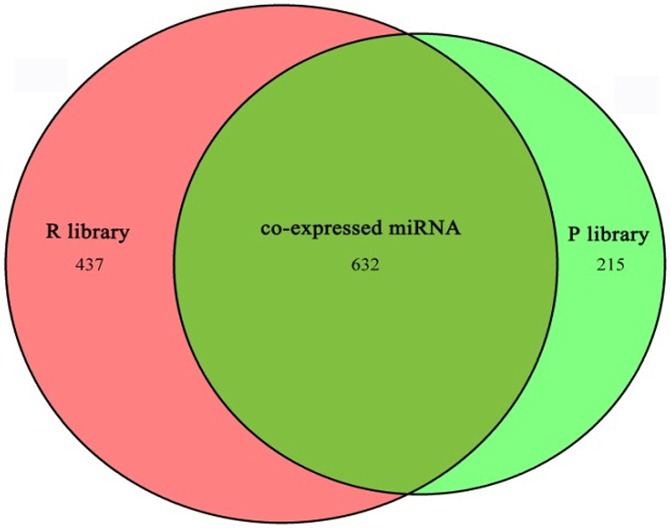
Venn diagrams of detected miRNAs.

The clean reads were subjected to advanced bioinformatic analyses and divided into four groups. (1) Group 1b, containing 14 miRNAs corresponding to 8 pre-miRNAs and miRNAs in miRBase 20.0; the pre-miRNAs further map to the genome and EST. (2) Group 2a, containing 215 miRNAs corresponding to 174 pre-miRNAs and miRNAs in miRBase 20.0; the mapped pre-miRNAs do not map to the genome, but the reads (including the miRNAs of the pre-miRNAs) map to the genome. The extended genome sequences from the genome loci may form hairpins. (3) Group 3a, containing 326 miRNAs corresponding to 270 pre-miRNAs and miRNAs in miRBase 20.0. The mapped pre-miRNAs and the reads do not map to the genome. (4) Group 4a, containing 621 miRNAs corresponding to 636 pre-miRNAs. Reads do not map to selected pre-miRNAs in miRbase 20.0, but do map to the genome. The extended genome sequences might form hairpins (Table 3). From this analysis, 1,284 miRNAs were identified, and 632 of these were co-expressed in both libraries, 1,069 miRNAs were detected in the R library, 847 were detected in the P library (Fig 2).

**Table 3:** Number of known miRNAs and novel miRNA candidates.

Groups:	Total		R library		P library	
	Pre-miRNA	Unique miRNA	Pre-miRNA	Unique miRNA	Pre-miRNA	Unique miRNA
gp1b	8	14	7	13	8	13
gp2a	174	215	162	200	154	193
gp3a	270	326	241	288	225	268
gp4a	636	621	485	470	293	280

Notes: gp1b: Reads map to selected (except for specific) miRNAs/pre-miRNAs in miRbase and the pre-miRNAs further map to the genome & EST. gp2a: Reads map to selected miRNAs/pre-miRNAs in miRbase. The mapped pre-miRNAs do not map to the genome, but the reads (and of course the miRNAs of the pre-miRNAs) map to genome. The extended genome sequences from the genome loci may form hairpins. gp3a: Reads map to slected miRNAs/pre-miRNAs in miRbase. The mapped pre-miRNAs do not map to the genome, and the reads do not map to the genome. gp4a: Reads do not map to selected pre-miRNAs in miRbase. But the reads map to genome & the extended genome sequences from genome may form hairpins.

### Identification of Known miRNAs

To identify known miRNAs in goat endometrium, the dataset was compared with the known mammalian miRNAs (miRNA precursors and mature miRNAs) in miRBase 20.0 (ftp://mirbase.org/pub/mirbase/CURRENT/). Sequences with a perfect match or one mismatch were retained in the alignment. We identified 545 known miRNAs in the R library and 522 in the P library (S1 Table). Considering the conservation of mature miRNAs among various species, the sequences of the existing miRNAs in goat were aligned and conservatively analyzed to investigate their evolutionary relationships (S2 Table).

The expression levels of oar-miR-10a_R+1_1ss12TA (a variant of oar-miR-10a as described previously in the same way [27]), oar-miR-10b_L+1R-1, oar-miR-26a, oar-miR-7a and aja-miR-143_1ss22GT were predominately higher, with more than 100,000 reads in both the R and P libraries (Table 4). These miRNAs constituted the most abundant read in the receptive endometrium, suggesting they potentially play an important role in the formation of receptive endometrium in goats. In addition, five known miRNAs (miR-449a, miR-182, miR-187-3p_R+1, miR-183-_L-1, miR-200a-5p) were detected by stem-loop qRT-PCR because they highly and differently expressed between P and R libraries in this study.

**Table 4:** Some known miRNA of which read number was more than 100,000 in this study.

miR name	miR seq	Rep_miR ID	R-NE	P-NE
oar-miR-10a_R+1_1ss12TA	TACCCTGTAGAACCGAATTTGT	oar-mir-10a	153958.5	657181.3
oar-miR-10b_L+1R-1	TACCCTGTAGAACCGAATTTGT	oar-mir-10b	160641.5	718168.0
oar-miR-26a	TTCAAGTAATCCAGGATAGGCT	oar-mir-26a	142846.7	330581.6
oar-let-7a	TGAGGTAGTAGGTTGTATAGTT	oar-let-7a	151415.4	223063.1
aja-miR-143_1ss22GT	TGAGATGAAGCACTGTAGCTCT	aja-mir-143	289634.3	1866274.0

Note: rep_miR ID was the representative known miRNA ID. R-NE represented the normalized expression level of miRNAs in the small RNA library generated from receptive endometrium of Xinong Saanen dairy goats. P-NE represented the normalized expression level of miRNAs in the small RNA library generated from pre-receptive endometrium of Xinong Saanen dairy goats.

### Identification of potential novel miRNAs

Sequencing reads that did not match any of the known miRNA were further analyzed to discover potential novel miRNAs. To determine whether these un-annotated small RNA reads were genuine miRNA, their hairpin structures, dicer cleavage sites and minimal free energies were explored using RNAfold software (http://rna.tbi.univie.ac.at/cgi-bin/RNAfold.cgi/). A total of 643 miRNA candidates were identified as having the typical stem-loop secondary structure, with 488 candidates identified in the R library and 298 in the P library (S3 Table). There were 33 mature miRNAs with NE numbers (Normalized expression) greater than 10 in the libraries (Table 5). The length of these miRNA sequences ranged from 18 nt to 24 nt. Moreover, 11 of 33 potential novel miRNA candidates were new 3p-derived sequences, and 22 were new 5p-derived sequences.

**Table 5:** Some predicted novel miRNA.

Index	miR_name	len	SS	R-NE	P-NE
1	PC-5p-69112_10	21	. . .(((..(((((((((..((((((((((((((((.((((..(. . . .)..)))).))))))))))))))))..)))))))))..))). . .	7.73	29.07
2–1	PC-5p-21385_42	22	. . .(((((((((((.((((((..(((((((((((((.((((. . . .)))))))))))))))))..)))))).)))))))).))). . .	7.74	13.96
2–2	PC-5p-21385_42	22	. . .((((((((((..(. . .(((..((((..((.(((.(((((((((.((((((..((((((. . . .. . . .. . . .))))))..)))))).))))))))).))))). . .))))..))). . .)..)))))). . . .)))). . .	7.74	13.96
3	PC-5p-104298_5	22	. . .(((((. . .. . .(((((((.((((((((((((((..(((((((..((((((. . .. . .)))))).)))))))..)))))))))))))).))))))). . .. . .)))))..	2.58	15.12
4–1	PC-5p-21767_42	22	. . .(((((((((((((((((((((.((((((((((((((. . .((((. . . ..))))..)))))))))))))).))))))))))))))))))))). . .	12.47	18.61
4–2	PC-5p-21767_42	22	. . .((((((.((((((..(((((((((((((((((((((((((((. . . .. . . .)))))))))))))))))))))))))))..)))))).)))))). . .	12.47	18.61
5–1	PC-5p-32617_26	22	. . .(..((((.(((((..((.(((((.((((((((((((..((. . .. . .))..)))))))))))).))))).))..))))).))))..). . .	5.16	12.79
5–2	PC-5p-32617_26	22	. . .((((((.(((((((.((((((..(((..((((((((((((((((.((. . . .. . . .))))))))))))))))))..)))..)))))).))))))))))))). . .	5.16	12.79
6	PC-3p-22067_41	19	. . .((((((((. . . .(((.((((. . .((((((. . . .. . .. . ..))))))..)))).)))..)))))))). . .	22.36	17.44
7	PC-3p-61234_13	23	. . .(((..(((((((((((((((((.((((. . . .((((((. . . .)))))). . . .)))).))))))))))))))))). . . ..))). . .	1.72	12.79
8	PC-5p-63323_12	19	. . .((((((((. . .((((((.((((((((. . . .. . . ..(((((((. . . .))))))))).))))..)).)))))))))))))). . .	10.32	19.77
9	PC-3p-25064_36	23	. . .(..((.((.((.((((((((((((.(((.((((.(((((((. . .((. . . ..)). . .))))))).))))))).))))))). . . .. . . .))))).)))).))..)..	36.11	1.16
10	PC-3p-99692_7	19	..((((((((. . .((((.(((.(((..((. . . ..)))))))).)))). . .))))))))..	11.18	2.33
11	PC-5p-39354_23	19	. . .(((((((.((. . .. . .(((.(((((.((((((..(((((. . . .)))))..)).)))).))))).))). . .)).)))).))). . .	11.18	13.96
12	PC-5p-60249_14	19	. . .((.((((((((((((.((. . . .((. . .((.(((((((. . .(((. . .(((. . .. . ..))). . .)))..))))))))). . .))..)))))).)))))))).)). . .	10.32	2.33
13	PC-5p-20958_45	22	. . .(((.(((((((((((((.(((((((((((((((((((((((. . .((. . .. . ..))..))))))))))))))))))))))).)))))))))))))..))). . .	28.38	17.44
14	PC-5p-26648_38	19	. . .(((((. . . .(((((((..((((((. . .. . ..)).)))). . .)))))))))))). . .	45.57	9.30
15	PC-3p-76502_8	22	. . .((((.(((..((((((((.((((((((((.((((. . . .. . . .. . . .. . . .. . . .)))).)))))))))).))))))))..))).)))). . .	17.19	31.40
16	PC-5p-6424_145	19	. . .(((((((((.((((((((..((((((. . . .. . . .. . .. . ..)))))). . . .)))))))).))))))))). . .	196.05	37.21
17	PC-5p-31071_29	23	..(((((((.((.(((((((((((((((. . .. . .((((. . . .. . . .))))))))))))))))))).)).)))))))..	13.76	17.44
18	PC-5p-29935_32	19	. . .(((((.(((((((. . .(((.((((((((. . . .. . .. . .)))))))).)))..))))..))).))))). . .	7.74	39.54
19	PC-3p-65381_11	21	.(((((((((((((.((((((((.((.(((((((((((. . . .. . .. . ..))))))))))).))..)))))))).))))))))))))).	13.76	8.14
20	PC-3p-42375_19	22	((.(((((((((.(((..(((((((.((. . .((((. . .)))))).)))))))..)))))))).)))).))	10.32	10.47
21	PC-3p-36269_24	20	. . .((((((((((((((.(((. . . ..(((((. . .(((((((. . .)))))))))))). . .))).))))))))).))))). . .	24.08	2.33
22	PC-5p-35677_26	19	((.(((((.(((((((((.((((((..(((..((. . .((((.((((((. . . .. . . .))))))))))))..)))..)))))).))))))))).))))).))	53.31	11.63
23	PC-5p-2673_284	23	.((. . .. . ..)).((((((((((. . . ..(((((.((((((. . . .(((. . .(..((((. . . .. . . .))))..). . .))). . .)))))).)))))..)))))))))). . .	3335	1146
24	PC-5p-10782_91	18	. . .((((((((((..((((((. . .((((..(.((. . . .)))..).)))))))))..))).)). . .))))). . .	65.35	25.58
25	PC-5p-196062_4	24	. . . .. . . .. . . .. . .. . ..(((((..((((((. . .(((..(((((((..(((. . . .)))..)))))))))).))))))..))))). . .	5.16	12.79
26	PC-3p-26002_37	21	. . .((((((((((((. . . .. . .. . .(((((((((.(((((. . . ..((. . . .(((. . .(((((((((. . .. . .)))..)))))). . .)))..))..))))).)))))..))))))))))..)))))). . .	122.1	106.9
27	PC-5p-66825_11	22	. . .(((((.(((..(((..((((..((((.((((((((.(((. . .. . .))))))))))).))))))))..)))..))).))))). . .	0	20.93
28	PC-5p-5933_158	18	. . . .. . . ..((((((. . .((((((..(((..(((.(((((.(((((.(..(.(. . . ..))..)))))).))))))))..)))..)))))). . .)))))). . .	164.23	13.96
29	PC-3p-61660_13	19	((.(((((. . .. . ..((((((. . . .(((.((..(..((. . . .((. . . .(((..((. . . ..))..))).))))..)..)).)))))))))..))))))).	17.20	8.14
30	PC-5p-335_2818	22	. . .((((((((((.((((((((((..((. . . .. . . .))))))))))))..)))))))))). . .	1831	2087
31	PC-5p-72586_9	22	. . .(((((((.(((..((((((((..(. . .)..))))))))..)))))))))). . .	2.58	11.63
32	PC-3p-16127_59	20	. . .(((((((((((..((. . .((((. . .((((((((. . . .. . . ..)))))))). . .))))..))..))))))))))). . .	45.57	12.79
33	PC-5p-20799_45	24	. . .(((.((.((((..(((((..(((((..((.(((((((((. . . .)).))))))))). . . .)))))..))))). . .. . .)))).)).))). . .	75.67	2.33

Note: Len represented the bases number of miRNA. SS represented secondary structure of predicted hairpin RNA (pre-miRNA) structures that were predicted with RNAfold software (http://rna.tbi.univie.ac.at/cgi-bin/RNAfold.cgi). R-NE represented the normalized expression level of miRNAs in the small RNA library generated from receptive endometrium of Xinong Saanen dairy goats. P-NE represented the normalized expression level of miRNAs in the small RNA library generated from pre-receptive endometrium of Xinong Saanen dairy goats.

**Fig 3:**
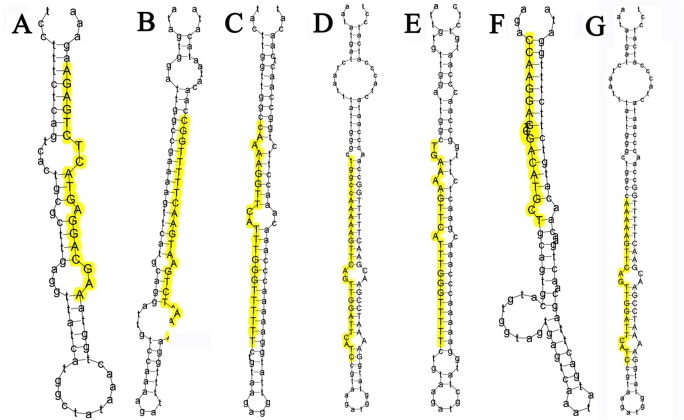
Stem-loop structures of 7 selected novel miRNAs in goat. Note: The secondary structures were generated from the goat genome and EST sequences. The uppercase letters refer to mature sequences and were indicated in yellow. A: PC-3p-22067_41, B: PC-3p-61234_13, C: PC-5p-21385_42, D: PC-5p-21767_42, E: PC-5p-32617_26, F: PC-5p-63323_12, G: PC-5p-104298_5.

The characteristic hairpin structure of miRNA precursors can be used to predict potential novel miRNA [28]. Therefore, the secondary structure of potential novel miRNAs was explored using RNAfold software, and the structures of 7 potential novel miRNA precursors (PC-3p-22067_41, PC-3p-61234_13, PC-5p-21385_42, PC-5p-21767_42, PC-5p-32617_26, PC-5p-63323_12 and PC-5p-104298_5) were predicted and showed in Fig 3. Similar to the results with the conserved miRNAs, the 7 sequences had significant stem-loop hairpin secondary structures. Moreover, ten novel miRNAs (PC-5p-5933_158, PC-5p-2673_284, PC-5p-6424_145, PC-5p-20799_45, PC-5p-35677_26, PC-3p-25064_36, PC-5p-26648_38, PC-3p-215602_2, PC-3p-82208_8, and PC-5p-108241_6) were selected due to their highly and differentially expressed levels between our two libraries.

### Differential expression of miRNAs in receptive and pre-receptive endometrium

**Fig 4:**
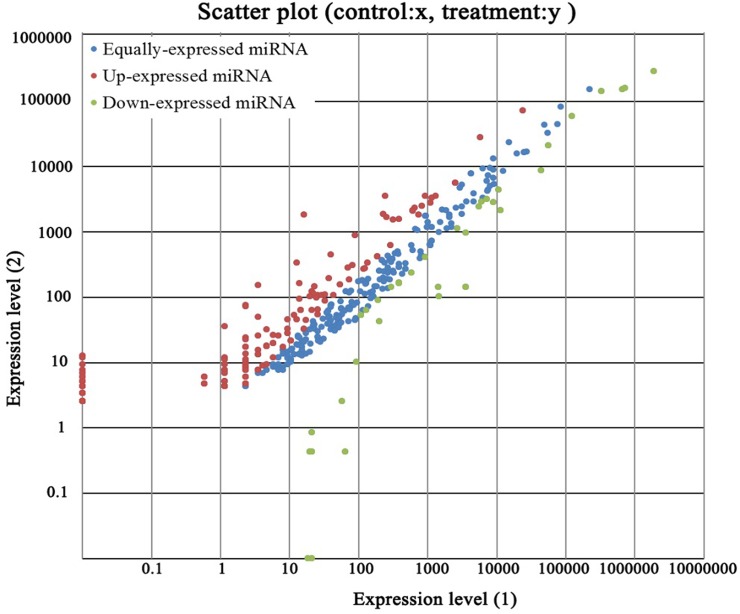
The differential expression levels of miRNAs between receptive and pre-receptive endometrium in goat. Note: Expression level (1): Expression level of pre-receptive endometrium; Expression level (2): Expression level of receptive endometrium. Each point in the Fig. represents a miRNA. Red points represent miRNAs with a fold change > 2, blue points represent miRNAs with 1/2 < fold change ≤ 2, green points represent miRNAs with fold change ≤ 1/2.

Pair-wise comparisons revealed important differentially expressed miRNAs between each developmental phase. Although some of their expression quantities were equivalent, there were some miRNAs expressed differently between the pre-receptive and receptive phases. We focused our attention on miRNAs meeting our designated criteria of *P*-values< 0.05 and |log2 (fold change)|> 1, and a total of 143 differentially expressed miRNAs were selected from the P and R libraries (Table 6). There were 33 differentially expressed miRNAs that were down-regulated in the receptive endometrium compared with pre-receptive endometrium in goats (fold change≤ 0.5), 231 miRNAs with fold changes were greater than 0.5 and less than 2 (0.5 <fold change≤ 2), and 110 miRNAs with fold changes greater than 2 (fold change> 2) (Fig 4). It deserved to note that there were some miRNAs expressed specifically, such as bta-miR-431_R-3 was detected only in the receptive endometrium and bta-miR-216a_R-2 in the pre-receptive endometrium (Table 6).

**Table 6:** Differential expression levels of miRNAs between R and P libraries.

Index	miR name	miR seq	R-NE	P-NE	log2 (R/P)	fold (R/P)	*P*-value
1	hsa-mir-7641-2-p5	ATCTCGGAAGCTAAGCAGGGTC	2334.57	657.07	1.83	3.55	0.00
2	oar-miR-22-3p_R+1	AAGCTGCCAGTTGAAGAACTGT	72245.41	23822.97	1.60	3.03	0.00
3	hsa-miR-34c-5p	AGGCAGTGTAGTTAGCTGATTGC	2777.84	1090.26	1.35	2.55	0.00
4	hsa-miR-34a-5p	TGGCAGTGTCTTAGCTGGTTGT	2522.03	830.34	1.60	3.04	0.00
5	hsa-miR-141-3p_R-1	TAACACTGTCTGGTAAAGATG	28337.35	5791.48	2.29	4.89	0.00
6	hsa-miR-449a	TGGCAGTGTATTGTTAGCTGGT	1842.72	16.28	6.82	113.18	0.00
7	hsa-mir-7641-2-p3_1ss22TC	GAAGCTAAGCAGGGTCGGGCCC	3564.20	929.19	1.94	3.84	0.00
8	bta-miR-31_R+2	AGGCAAGATGCTGGCATAGCTGT	3547.01	1305.99	1.44	2.72	0.00
9	hsa-miR-141-3p_R+1	TAACACTGTCTGGTAAAGATGGC	1673.33	252.36	2.73	6.63	0.00
10	cgr-miR-142-5p_L-2R+1	CATAAAGTAGAAAGCACTACT	5689.25	2523.59	1.17	2.25	0.00
11	bta-miR-182	TTTGGCAATGGTAGAACTCACACT	3518.63	241.89	3.86	14.55	0.00
12	cgr-miR-200c	TAATACTGCCGGGTAATGATGGA	1555.52	316.32	2.30	4.92	0.00
13	ptr-miR-203_L-1R+1	TGAAATGTTTAGGACCACTAGT	1896.90	230.26	3.04	8.24	0.00
14	bta-miR-204	TTCCCTTTGTCATCCTATGCCT	2120.46	608.22	1.80	3.49	0.00
15	hsa-miR-204-5p	TTCCCTTTGTCATCCTATGCCT	2116.17	607.06	1.80	3.49	0.00
16	bta-miR-339a_R+1	TCCCTGTCCTCCAGGAGCTCACT	1836.27	736.73	1.32	2.49	0.00
17	cfa-miR-375	TTTGTTCGTTCGGCTCGCGTGA	882.24	89.55	3.30	9.85	0.00
18	mmu-miR-6240_R-8_1ss1CA	ACAAAGCATCGCGAAGGC	1583.04	381.45	2.05	4.15	0.00
19	PC-5p-2673_284	ACAGGATTGACAGATTGATAGCT	3335.48	1146.67	1.54	2.91	0.00
20	hsa-miR-200a-5p	CATCTTACCGGACAGTGCTGGA	450.58	39.54	3.51	11.40	0.00
21	hsa-miR-187-3p_R+1	TCGTGTCTTGTGTTGCAGCCGGT	338.79	12.79	4.73	26.48	0.00
22	hsa-miR-17-5p	CAAAGTGCTTACAGTGCAGGTAG	619.97	293.06	1.08	2.12	0.00
23	cgr-miR-142-3p_L+1R+1	TGTAGTGTTTCCTACTTTATGGA	423.49	185.49	1.19	2.28	0.00
24	hsa-mir-7641-2-p3_1ss1TC	CGGTTAGTACTTGGATGGGA	311.28	81.41	1.93	3.82	0.00
25	bta-miR-6529	GAGAGATCAGAGGCGCAGAGT	281.18	69.78	2.01	4.03	0.00
26	hsa-miR-190a-5p_R+1	TGATATGTTTGATATATTAGGTT	335.35	133.74	1.33	2.51	0.00
27	bta-miR-183_L-1	ATGGCACTGGTAGAATTCACTG	153.92	3.49	5.46	44.12	0.00
28	PC-5p-5933_158	CTGTACCACCTTGTCGGG	164.24	13.96	3.56	11.77	0.00
29	PC-5p-6424_145	CAGCCTCTGGCATGTTGGA	196.05	37.21	2.40	5.27	0.00
30	bta-miR-132	TAACAGTCTACAGCCATGGTCG	271.72	117.46	1.21	2.31	0.00
31	bta-miR-15b	TAGCAGCACATCATGGTTTACA	274.30	125.02	1.13	2.19	0.00
32	hsa-miR-4792_L+1R+1_1ss10GT	CCGGTGAGCTCTCGCTGGCC	146.18	23.26	2.65	6.28	0.00
33	hsa-miR-31-3p_R+1	TGCTATGCCAACATATTGCCATC	188.31	73.27	1.36	2.57	0.00
34	hsa-miR-4454_L+1_1ss3GA	CGAATCCGAGTCACGGCACCA	122.96	20.93	2.55	5.87	0.00
35	bta-miR-210_L-1R+1	CTGTGCGTGTGACAGCGGCTGAT	156.50	53.50	1.55	2.93	0.00
36	mmu-miR-22-5p_R-1	AGTTCTTCAGTGGCAAGCTTT	116.08	22.10	2.39	5.25	0.00
37	PC-5p-20799_45	GAGGGTTTGGGTTTGGTCGTGGGA	75.67	2.33	5.02	32.53	0.00
38	mmu-miR-3963_R+2_1ss1TC	CGTATCCCACTTCTGACACCA	102.33	19.77	2.37	5.18	0.00
39	hsa-miR-132-5p	ACCGTGGCTTTCGATTGTTACT	93.73	13.96	2.75	6.72	0.00
40	hsa-miR-184	TGGACGGAGAACTGATAAGGGT	72.23	2.33	4.96	31.05	0.00
41	rno-miR-147_R-1	GTGTGCGGAAATGCTTCTGCT	106.63	25.58	2.06	4.17	0.00
42	oar-miR-758-3p_R+2	TTTGTGACCTGGTCCACTAACT	106.63	29.07	1.87	3.67	0.00
43	cgr-miR-32-5p	TATTGCACATTACTAAGTTGC	109.20	32.56	1.75	3.35	0.00
44	bta-miR-2478_L+2	TCGTATCCCACTTCTGACACCA	98.89	24.42	2.02	4.05	0.00
45	hsa-miR-24-1-5p_L+1_3ss17TA19TC20-A	GTGCCTACTGAGCTGAAACACAGT	108.34	43.03	1.33	2.52	0.00
46	oan-miR-1386_L+4	TCGGCTCCTGGCTGGCTCGCCA	87.71	32.56	1.43	2.69	0.00
47	oan-miR-429-3p_R+2	TAATACTGTCTGGTAATGCCGTAA	49.87	3.49	3.84	14.30	0.00
48	hsa-miR-9-3p_L-1R+1	TAAAGCTAGATAACCGAAAGTA	63.63	14.54	2.13	4.38	0.00
49	ggo-miR-203b	TTGAACTGTTAAGAACCACTGG	63.63	20.93	1.60	3.04	0.00
50	PC-5p-35677_26	CTCCGTCCCGGGACCCGGG	53.31	11.63	2.20	4.58	0.00
51	PC-3p-25064_36	TGACTTCCCCCTGTCCACTCAGT	36.11	1.16	4.96	31.05	0.00
52	PC-5p-10782_91	CTTGACTCTAGTCTGGCA	65.35	25.58	1.35	2.55	0.00
53	PC-5p-26648_38	TCCCGGGGCCGAGGGAGCC	45.57	9.30	2.29	4.90	0.00
54	PC-3p-16127_59	AAGGAAAATGTTCTTATTTT	45.57	12.79	1.83	3.56	0.00
55	hsa-miR-153-3p	TTGCATAGTCACAAAAGTGATC	54.17	25.58	1.08	2.12	0.00
56	bta-miR-96	TTTGGCACTAGCACATTTTTGCT	31.82	4.65	2.77	6.84	0.00
57	hsa-miR-26b-3p	CCTGTTCTCCATTACTTGGCTC	44.71	17.44	1.36	2.56	0.00
58	mmu-miR-3968_1ss14AT	CGAATCCCACTCCTGACACCA	25.80	3.49	2.89	7.39	0.00
59	hsa-miR-34c-3p	AATCACTAACCACACGGCCAGG	32.68	9.30	1.81	3.51	0.00
60	PC-3p-36269_24	CGCCCCACCCCGCTGCGGGC	24.08	2.33	3.37	10.35	0.00
61	hsa-miR-21-5p_R+2_1ss4CN	TAGNTTATCAGACTGATGTTGACC	22.36	2.33	3.26	9.61	0.00
62	mmu-miR-503-5p	TAGCAGCGGGAACAGTACTGCAG	26.66	5.81	2.20	4.58	0.00
63	bta-miR-2285f_L+1R-1	AAAAACCTGAATGAACTTTTTG	25.80	6.98	1.89	3.70	0.00
64	bta-miR-6123_R+1	TGCCAAGCCCACGTTCAAAGGC	28.38	9.30	1.61	3.05	0.00
65	ppy-miR-1260b_R+2_1ss9AG	ATCCCACCGCTGCCACCATT	32.68	16.28	1.00	2.01	0.00
66	bta-miR-212	ACCTTGGCTCTAGACTGCTTACT	17.20	2.33	2.89	7.39	0.00
67	hsa-miR-212-5p	ACCTTGGCTCTAGACTGCTTACT	17.20	2.33	2.89	7.39	0.00
68	hsa-miR-212-3p	TAACAGTCTCCAGTCACGGCC	19.78	5.81	1.77	3.40	0.00
69	cgr-miR-32-3p	CAATTTAGTGTGTGTGATATT	18.06	4.65	1.96	3.88	0.00
70	hsa-miR-340-3p_R+1	TCCGTCTCAGTTACTTTATAGCC	17.63	4.65	1.92	3.79	0.00
71	bta-miR-340	TCCGTCTCAGTTACTTTATAGCC	17.63	4.65	1.92	3.79	0.00
72	bta-miR-2427_L+1R-1	TAGGTCATTTCAAAGAGGGCT	13.76	2.33	2.56	5.92	0.00
73	hsa-miR-190a-3p_L+1	ACTATATATCAAACATATTCCT	15.48	3.49	2.15	4.44	0.00
74	oar-miR-154b-3p_L-1	ATCATACATGGTTGACCTTTTT	12.04	1.16	3.37	10.35	0.00
75	bta-miR-545-3p_L-1R+1	TCAACAAACATTTATTGTGTGC	21.50	10.47	1.04	2.05	0.00
76	hsa-miR-141-5p_1ss11TC	CATCTTCCAGCACAGTGTTGGA	11.18	1.16	3.26	9.61	0.00
77	hsa-miR-491-3p	CTTATGCAAGATTCCCTTCTAC	13.33	3.49	1.93	3.82	0.00
78	PC-3p-61660_13	CGCCTGTCTGAGCCTCGCT	17.20	8.14	1.08	2.11	0.00
79	PC-3p-99692_7	TCCGCCGGCCTTGCGGGCC	11.18	2.33	2.26	4.81	0.00
80	PC-5p-91245_7	CGCGCGCGGGGCCCGGGG	9.46	1.16	3.02	8.13	0.00
81	PC-5p-60249_14	ATTCCTATAATTCTAGCCA	10.32	2.33	2.15	4.44	0.00
82	PC-3p-215602_2	TGCAAAGAGGTTGGATCGAGT	7.74	1.16	2.73	6.65	0.00
83	PC-3p-82208_8	AAGGAGGTGCTGGTTGCTTT	7.74	1.16	2.73	6.65	0.00
84	bta-mir-2285o-3-p5_1ss12TC	AAAAATTTGTTCGGGTTTTTCT	9.46	2.33	2.02	4.07	0.00
85	hsa-miR-139-3p	TGGAGACGCGGCCCTGTTGGAGT	8.60	2.33	1.89	3.70	0.00
86	cgr-miR-139-3p	TGGAGACGCGGCCCTGTTGGAGT	8.60	2.33	1.89	3.70	0.00
87	bta-miR-2284ab	TAAAAGTTTGGTTGGGTTTTT	12.04	5.81	1.05	2.07	0.00
88	PC-5p-108241_6	ATACCGGGTGCTAGGCTT	6.88	1.16	2.56	5.92	0.00
89	mdo-miR-22-3p_R+1	AAGCTGCCAGTTGAAGAACTGCA	7.74	2.33	1.73	3.33	0.00
90	hsa-miR-185-3p_L+1R-1_1ss22TC	CAGGGGCTGGCTTTCCTCTGGC	7.74	2.33	1.73	3.33	0.00
91	eca-miR-219-5p_R+2	TGATTGTCCAAACGCAATTCTCG	9.03	4.07	1.15	2.22	0.00
92	hsa-miR-26a-1-3p_1ss9TC	CCTATTCTCGGTTACTTGCACG	7.74	3.49	1.15	2.22	0.00
93	hsa-miR-122-5p_R-1	TGGAGTGTGACAATGGTGTTT	7.74	3.49	1.15	2.22	0.00
94	PC-5p-139329_4	GGGTGCTGATAGTGAGGC	7.74	3.49	1.15	2.22	0.00
95	bta-miR-2284d_L-1R+1_1ss10GA	AAAAGTTCATTAGGGTTTTTCT	6.02	0.58	3.37	10.35	0.00
96	bta-mir-2284z-4-p5_1ss1AG	GAAAGTTTGTTTGGGTTTTTCT	9.46	4.65	1.02	2.03	0.00
97	bta-mir-2887-2-p5_1ss7AC	CGGGACCCGGGGCGCGGC	6.02	2.33	1.37	2.59	0.00
98	bta-miR-677_R+1	CTCACTGATGAGCAGCTTCTGACT	6.02	2.33	1.37	2.59	0.00
99	hsa-miR-153-5p_L+1_2ss15G-16TC	GTCATTTTTGTGATCTGCAGCT	5.16	1.16	2.15	4.44	0.01
100	bta-miR-2285r_R-2	AGAAACCTGGATGAACTTTTT	5.16	1.16	2.15	4.44	0.01
101	hsa-miR-628-3p_L+1	TTCTAGTAAGAGTGGCAGTCGA	4.73	0.58	3.02	8.13	0.01
102	hsa-miR-138-5p	AGCTGGTGTTGTGAATCAGGCCG	4.73	2.33	1.02	2.03	0.01
103	hsa-miR-4791_1ss17AT	TGGATATGATGACTGATA	4.73	2.33	1.02	2.03	0.01
104	hsa-miR-377-3p	ATCACACAAAGGCAACTTTTGT	4.30	1.16	1.89	3.70	0.02
105	ssc-miR-671-5p	AGGAAGCCCTGGAGGGGCTGGAGG	4.30	1.16	1.89	3.70	0.02
106	bta-miR-671_R+1	AGGAAGCCCTGGAGGGGCTGGAGG	4.30	1.16	1.89	3.70	0.02
107	PC-5p-203577_2	AAGAAGTTCATTCGGGTTTTTC	4.30	1.16	1.89	3.70	0.02
108	PC-5p-94605_6	GCTGGCCTGGAGCCGGGCG	4.30	1.16	1.89	3.70	0.02
109	PC-3p-247920_2	CTGGATATCTGAGACTCAGTTT	4.30	1.16	1.89	3.70	0.02
110	PC-5p-125235_4	GCCAAAAAGTTTGTTTGGGCTTT	4.30	1.16	1.89	3.70	0.02
111	oar-miR-10a_R+1_1ss12TA	TACCCTGTAGAACCGAATTTGT	153958.53	657181.33	0.23	-2.09	0.00
112	oar-miR-10b_L+1R-1	TACCCTGTAGAACCGAATTTGT	160641.52	718168.02	0.22	-2.16	0.00
113	oar-miR-26b_R+1	TTCAAGTAATTCAGGATAGGTT	59229.84	124009.11	0.48	-1.07	0.00
114	oar-miR-26a	TTCAAGTAATCCAGGATAGGCT	142846.73	330581.63	0.43	-1.21	0.00
115	cgr-miR-101b-3p_1ss22GT	TACAGTACTGTGATAACTGAAT	8689.09	43584.93	0.20	-2.33	0.00
116	aja-miR-143_1ss22GT	TGAGATGAAGCACTGTAGCTCT	289634.30	1866274.04	0.16	-2.69	0.00
117	oar-miR-133_L+1	TTTGGTCCCCTTCAACCAGCTGT	144.89	3558.04	0.04	-4.62	0.00
118	cfa-miR-145	GTCCAGTTTTCCCAGGAATCCCT	2123.04	11338.73	0.19	-2.42	0.00
119	bta-miR-490_R+1	CAACCTGGAGGACTCCATGCTGT	103.19	1465.31	0.07	-3.83	0.00
120	bta-miR-335_R-1	TCAAGAGCAATAACGAAAAATG	143.60	1430.42	0.10	-3.32	0.00
121	cfa-miR-196b	TAGGTAGTTTCCTGTTGTTGGGA	2885.76	5979.87	0.48	-1.05	0.00
122	mdo-miR-181b-5p	AACATTCATTGCTGTCGGTGGGT	3169.52	7213.76	0.44	-1.19	0.00
123	oar-miR-181a	AACATTCAACGCTGTCGGTGAGT	20920.02	55420.24	0.38	-1.41	0.00
124	hsa-miR-181c-5p_R+1	AACATTCAACCTGTCGGTGAGTT	4420.64	10611.89	0.42	-1.26	0.00
125	hsa-miR-192-5p	CTGACCTATGAATTGACAGCC	2454.10	5510.04	0.45	-1.17	0.00
126	hsa-miR-28-3p_1ss11TA	CACTAGATTGAGAGCTCCTGGA	972.52	3577.22	0.27	-1.88	0.00
127	oar-miR-133_L+1R-2	TTTGGTCCCCTTCAACCAGCT	0.43	64.54	0.01	-7.23	0.00
128	ptr-miR-92_R+2	TATTGCACTTGTCCCGGCCTGTAA	1121.86	2665.86	0.42	-1.25	0.00
129	hsa-miR-100-5p	AACCCGTAGATCCGAACTTGTG	416.18	915.24	0.45	-1.14	0.00
130	hsa-miR-767-5p_R-3	TGCACCATGGTTGTCTGAGC	2.58	56.98	0.05	-4.47	0.00
131	hsa-miR-490-5p	CCATGGATCTCCAGGTGGGT	10.32	93.04	0.11	-3.17	0.00
132	bta-miR-320a	AAAAGCTGGGTTGAGAGGGCGA	2877.59	9040.17	0.32	-1.65	0.00
133	hsa-miR-299-3p_1ss10TC	TATGTGGGACGGTAAACCGCTT	141.88	297.71	0.48	-1.07	0.00
134	hsa-miR-133a-5p	AGCTGGTAAAATGGAACCAAAT	0.43	19.77	0.02	-5.52	0.00
135	hsa-miR-335-3p	TTTTTCATTATTGCTCCTGACC	42.99	198.86	0.22	-2.21	0.00
136	hsa-miR-1_1ss1TC	CGGAATGTAAAGAAGTATGTAT	0.43	20.93	0.02	-5.61	0.00
137	age-miR-19a_1ss23AT	TGTGCAAATCTATGCAAAACTGT	90.29	190.72	0.47	-1.08	0.02
138	hsa-miR-574-5p_R-2	TGAGTGTGTGTGTGTGAGTGT	166.67	386.68	0.43	-1.21	0.02
139	hsa-miR-374a-3p_R-1_1ss9AG	CTTATCAGGTTGTATTGTAAT	164.24	382.61	0.43	-1.22	0.02
140	hsa-miR-143-5p_1ss22TA	GGTGCAGTGCTGCATCTCTGGA	238.19	576.82	0.41	-1.28	0.02
141	mdo-miR-599-5p_R-2_1ss9TG	TTTGATAAGCTGACATGGGAC	0.86	20.93	0.04	-4.61	0.02
142	hsa-miR-499a-5p	TTAAGACTTGCAGTGATGTTT	63.63	130.25	0.49	-1.03	0.03
143	bta-mir-2285m-3-p5_1ss1AG	GAAAGGTTCATTTGGGTTTTT	53.74	108.15	0.50	-1.01	0.03
144	bta-miR-216a_R-2	TAATCTCAGCTGGCAACTGT	0	18.61	-	-	-
145	bta-miR-431_R-3	TGTCTTGCAGGCCGTCATGC	12.90	0	-	-	-

Note: R-NE represented the normalized expression level of miRNAs in the small RNA library generated from receptive endometrium of Xinong Saanen dairy goats. P-NE represented the normalized expression level of miRNAs in the small RNA library generated from pre-receptive endometrium of Xinong Saanen dairy goats. Fold (R/P) and log2 (R/P) indicated the fold change of the miRNAs between samples. P-Value manifested the significance of miRNAs differential expression between two samples.

The most differentially expressed miRNA was hsa-miR-449a, which had a 113.2-fold (NE> 1,000) increase in the receptive endometrium compared with the pre-receptive endometrium. Other miRNA that were differentially expressed between our two libraries included bta-miR-183_L-1, PC-5p-20799_45, hsa-miR-184, PC-3p-25064_36, hsa-miR-187-3p_R+1, oan-miR-429-3p_R+2, PC-5p-5933_158, hsa-miR-200a-5p, PC-3p-36269_24, oar-miR-154b-3p_L-1 and so on (Table 6). Moreover, bta-miR-182 had the high expression level (NE = 3518.63) and increased 14.55-fold in R library compare with P library (Table 7).

**Table 7:** Some differential expressed miRNAs (|log2 (fold change)|> 3) and their targets.

Index	miR_name	Fold (R/P)	log2 (R/P)	R-NE	P-NE	Number of Targets
1	hsa-miR-449a	113.18	6.82	1842.72	16.28	1030
2	bta-miR-183_L-1	44.12	5.46	153.92	3.49	712
3	PC-5p-20799_45	32.53	5.02	75.67	2.33	557
4	hsa-miR-184	31.05	4.96	72.23	2.33	273
5	PC-3p-25064_36	31.05	4.96	36.11	1.16	829
6	hsa-miR-187-3p_R+1	26.48	4.73	338.79	12.79	352
7	bta-miR-182	14.55	3.86	3518.63	241.89	756
8	oan-miR-429-3p_R+2	14.3	3.84	49.87	3.49	679
9	PC-5p-5933_158	11.77	3.56	164.24	13.96	427
10	hsa-miR-200a-5p	11.4	3.51	450.58	39.54	351
11	PC-3p-36269_24	10.35	3.37	24.08	2.33	806
12	oar-miR-154b-3p_L-1	10.35	3.37	12.04	1.16	444
13	bta-miR-2284d_L-1R+1_1ss10GA	10.35	3.37	6.02	0.58	515
14	cfa-miR-375	9.85	3.3	882.24	89.55	220
15	hsa-miR-141-5p_1ss11TC	9.61	3.26	11.18	1.16	988
16	ptr-miR-203_L-1R+1	8.24	3.04	1896.9	230.26	789
17	PC-5p-91245_7	8.13	3.02	9.46	1.16	81
18	hsa-miR-628-3p_L+1	8.13	3.02	4.73	0.58	325
19	oar-miR-133_L+1R-2	0.01	-7.23	0.43	64.54	471
20	hsa-miR-133a-5p	0.02	-5.52	0.43	19.77	649
21	hsa-miR-1_1ss1TC	0.02	-5.61	0.43	20.93	613
22	oar-miR-133_L+1	0.04	-4.62	144.89	3558.04	956
23	mdo-miR-599-5p_R-2_1ss9TG	0.04	-4.61	0.86	20.93	406
24	hsa-miR-767-5p_R-3	0.05	-4.47	2.58	56.98	626
25	bta-miR-490_R+1	0.07	-3.83	103.19	1465.31	813
26	bta-miR-335_R-1	0.1	-3.32	143.6	1430.42	765
27	hsa-miR-490-5p	0.11	-3.17	10.32	93.04	649

Note: R-NE represented the normalized expression level of miRNAs in the small RNA library generated from receptive endometrium of Xinong Saanen dairy goats. P-NE represented the normalized expression level of miRNAs in the small RNA library generated from pre-receptive endometrium of Xinong Saanen dairy goats. Fold (R /P) and log2 (R/P) indicated the fold change of the miRNAs between samples. *P*-Value manifested the significance of miRNAs differential expression between two samples. Number of Targets represented putative target genes for a certain miRNA predicted by two software programs (Target Scan v.50 and Mireap v.3.3a).

### Target gene predictions for differentially expressed miRNAs

For animal miRNAs, sequence complementarities with their targets were usually restricted to the 5’ region, particularly those at nucleotide positions 2 through 7, and it was likely that many miRNAs function through cooperative regulation of multiple mRNAs [20]. Owing to the imperfect complementarity of animal miRNAs with their targets, it is more challenging to identify targets and difficult to judge the accuracy of the prediction. Thus, two software programs (Target Scan v.50 and Mireap v.3.3a) were used to predict target genes for the differentially expressed miRNAs. As a result, a total of 7,608 annotated mRNA transcripts were predicted as putative target genes for the 143 differentially expressed miRNAs (S4 Table). One miRNA can target multiple genes, for example, 1,030 annotated mRNA transcripts were predicted as putative targets for hsa-miR-449a, which the most differentially expressed miRNA, and 756 putative targets for bta-miR-182 (Table 7). Moreover, our results indicated that some genes were regulated by more than one miRNA, such as JR117835.1 that was targeted by 8 miRNAs (S4 Table).

### GO enrichment and KEGG pathway analysis

We used a well-established miRNA-target dataset to investigate the possible function of these miRNAs with the aid of gene function annotation methods, including Gene Ontology (GO) and Kyoto Encyclopedia of Genes and Genomes (KEGG) progress.

**Fig 5:**
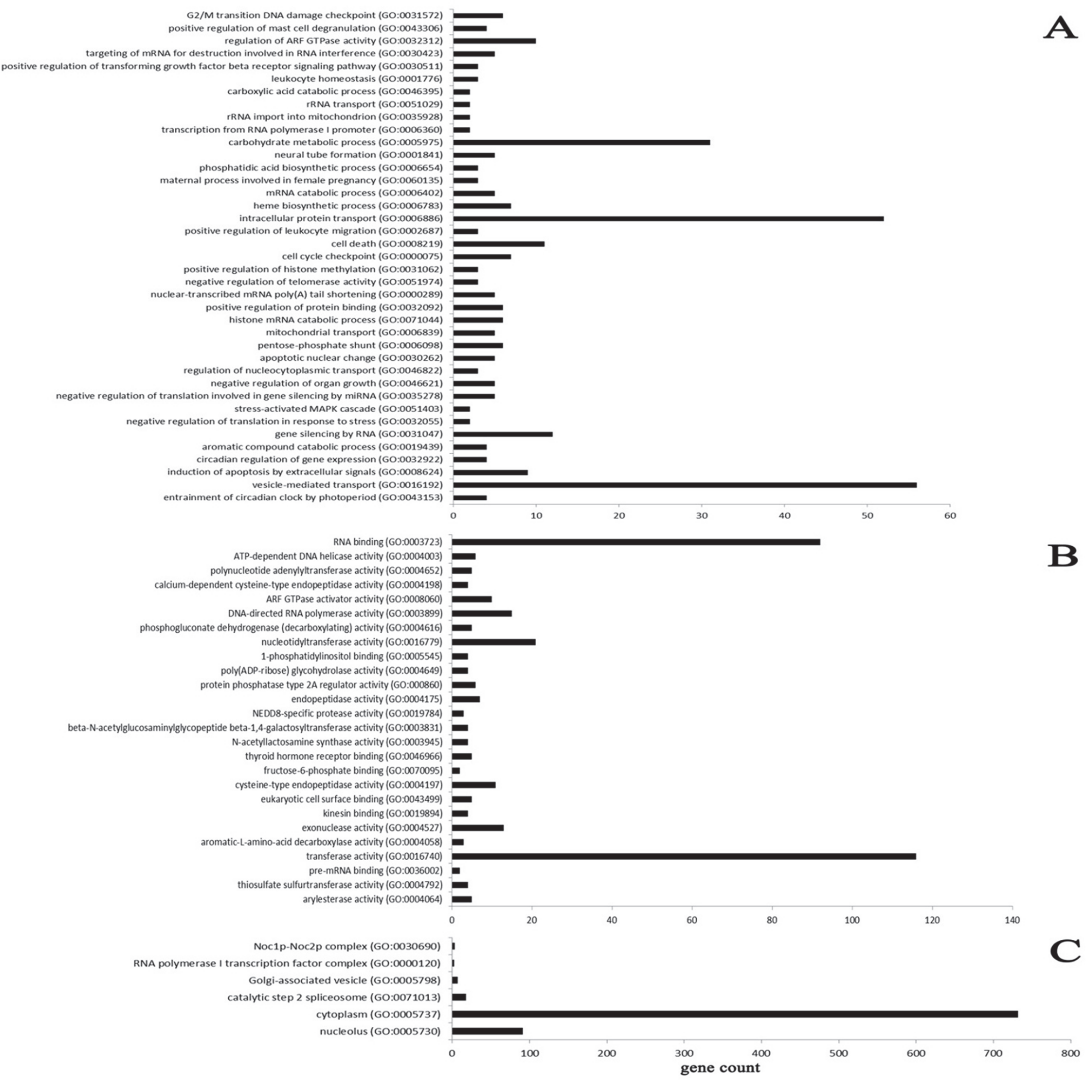
The GO enrichment analysis of predicted targets of differentially expressed miRNAs with *P*< 0.05. Note: A: Biological process, B: Molecular function, C: Cellular component. The GO terms were sorted by the enrichment p-value calculated by the calculating formula:
$$\text{P}=1-\overset{\text{m}-1}{\underset{\text{i }=0}{\sum }}\frac{\left(\frac{\text{M}}{\text{i}}\right)\left(\frac{\text{N}-\text{M}}{\text{n}-\text{i}}\right)}{\frac{\text{N}}{\text{n}}}$$
The N represented the number of GO annotated genes in genome, n represented the number of differentially expressed genes in N. M represented the number of particular GO annotated genes in genome, m represented the number of particular GO annotated genes expressed differentially in M.

**Fig 6:**
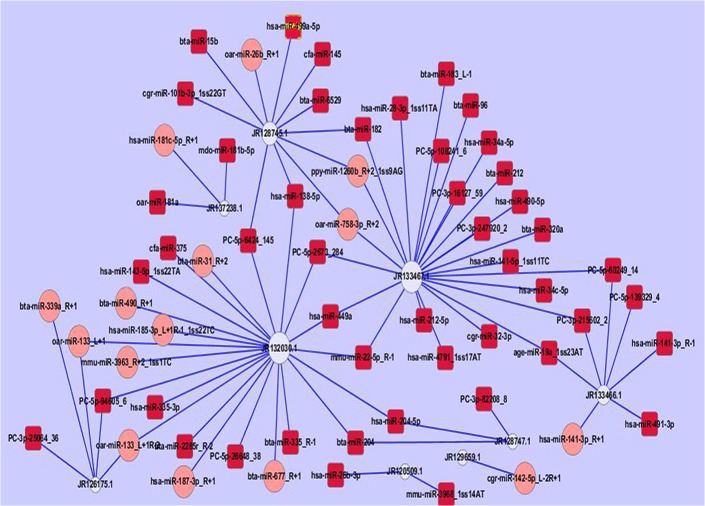
miRNA-gene network of induction of apoptosis by extracellular signals (GO: 0008624). Note: White circular nodes represent genes, and red rectangular and pink rounded rectangle nodes represent miRNAs. The size of the nodes represents the power of the interrelation among the nodes, and edges between two nodes represent interactions between genes. The more edges a gene has, the more miRNAs that interact with it, and the more central a role it had within the network. The top five key miRNAs in the network were miR-449a, miR-182, PC-5p-6424_145, PC-5p-2673_284, and miR-138-5p. The top three key mRNAs’ serial numbers were JR128745.1 (Caspase 8, apoptosis-related cysteine peptidase (CASP8)), JR133467.1 (Y-linked ubiquitin-specific protease 9 (USP9Y)), and JR132030.1 (CD5 molecule (CD5)) in NCBI (National Center for Biotechnology Information).

GO enrichment analysis using cellular components showed that 490 genes (13.64%) mapped to GO terms for component topology in the database. The analysis of biological processes showed that 2,087 genes (58.10%) were involved in cellular or metabolic processes, and the analysis of molecular function showed that 1,015 genes (28.26%) were assigned different functions based on gene background (Fig 5 and S5 Table). GO biological process analysis based on the predicted targets showed that the differentially expressed miRNAs were involved in remodeling of the receptive endometrium, including induction of apoptosis by extracellular signals (GO: 0008624) (Fig 6). Other miRNA-gene networks of interest included cell cycle checkpoints (GO: 0000075), vesicle-mediated transport (GO: 0016192), intracellular protein transport (GO: 0006886) and maternal processes involved in female pregnancy (GO: 0060135).

**Fig 7:**
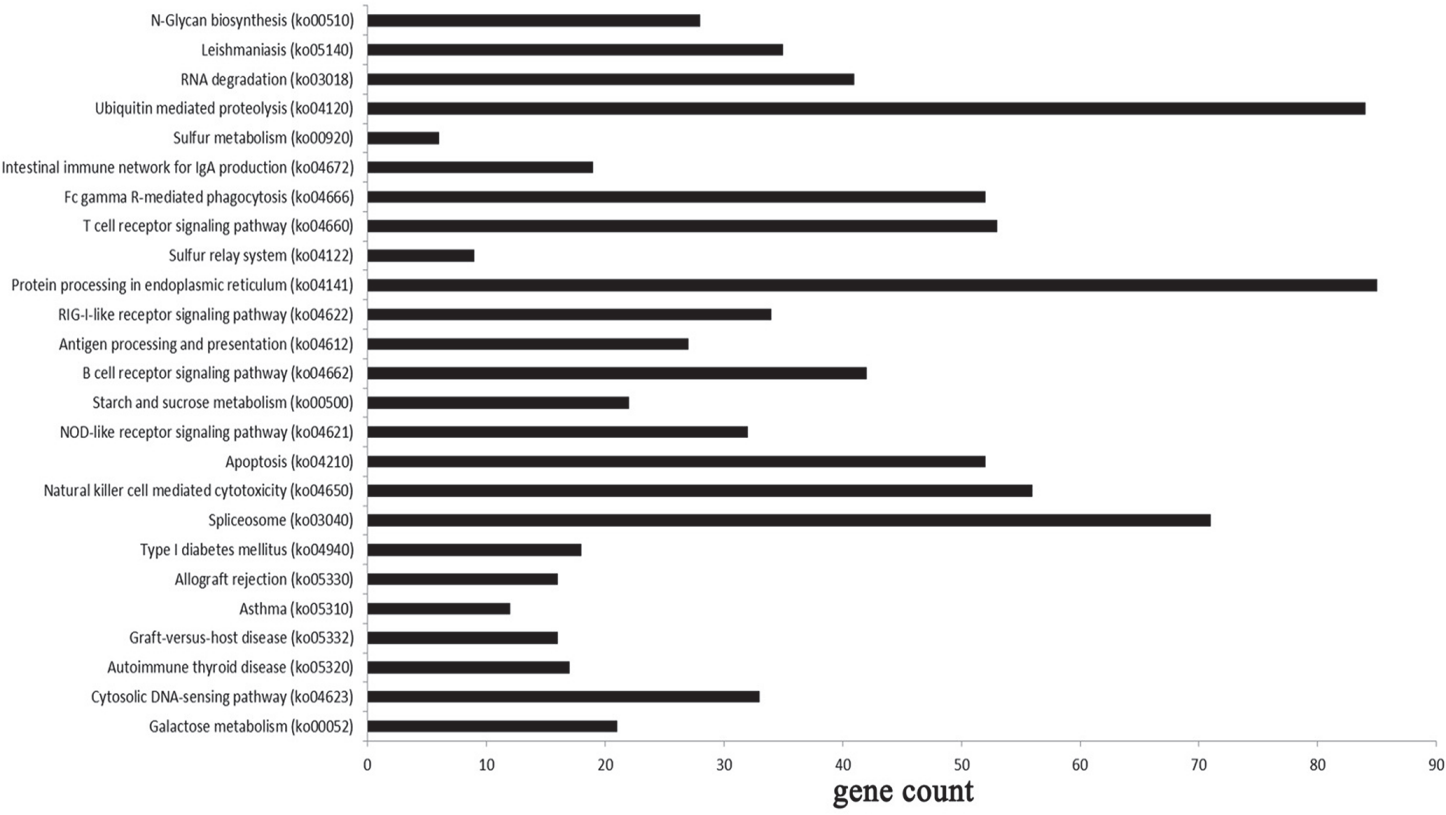
The KEGG pathway analysis of predicted targets differentially expressed miRNAs with *P*< 0.05. Note: The enrichment p-value calculated by the calculating formula:
$$P=1-\overset{m-1}{\underset{i=0}{\sum }}\frac{\left(\frac{M}{i}\right)\left(\frac{N-M}{n-i}\right)}{\frac{N}{n}}$$
N was the number of all genes with KEGG annotation, *n* was the number of target gene candidates in N, M was the number of all genes annotated to a certain pathway, *m* was the number of target gene candidates in M.

KEGG pathway annotation showed 1,886 target genes that were annotated for 220 biological processes (S6 Table). Moreover, there were 25 KEGG pathways with P values less than 0.05 (Fig 7). KEGG pathway analysis based on predicted targets revealed that differentially expressed miRNAs were involved in several pathways affecting the development of endometrium, including ubiquitin mediated proteolysis (84 annotated genes), T cell receptor signaling pathway (53 annotated genes), Fc gamma R-mediated phagocytosis (52 annotated genes) and apoptosis (52 annotated genes) (S6 Table).

### Validation of miRNA expression with Stem-loop qRT-PCR

**Fig 8:**
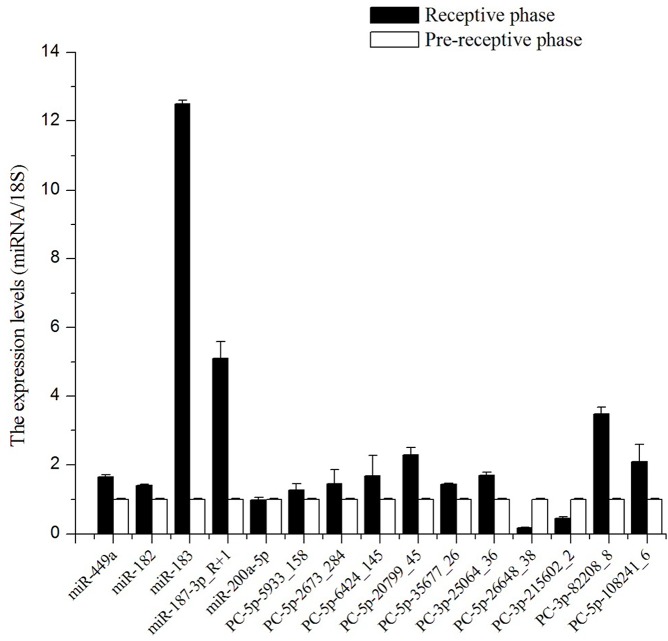
The expression levels of 15 miRNAs were determined by Stem-loop RT-qPCR. Note: *18S* was used as internal control gene for normalization in our experiments. Values were “means ± SD” in receptive endometrium (n = 9) that were relative to pre-receptive endometrium (n = 9), whose values all were 1 in this manuscript because of the method of 2^-ΔΔCt^, ΔΔ Ct = (CT _miRNA_ − CT _18s_) _R_ − (CT _miRNA_ − CT _18s_) _P_.

In general, qRT-PCR is considered the standard method to detect gene expression. To validate the reliability of the sequencing data, we applied Stem-loop RT-qPCR to compare the expression levels of the differentially expressed miRNAs and newly identified miRNAs. Five conserved miRNAs (miR-449a, miR-182, miR-200a-5p, miR-187-3p_R+1, miR-183_L-1, miR-216a_R-2 and miR-431_R-3) and ten potential novel miRNAs (PC-5p-6424_145, PC-5p-2673_284, PC-5p-5933_158 PC-5p-20799_45, PC-5p-35677_26, PC-3p-25064_36, PC-3p-82208_8, PC-5p-108241_6, PC-5p-26648_38 and PC-3p-215602_2) were selected due to these miRNAs differential expression between R and P libraries, and may participate in the formation of endometrial receptivity. The expression levels of twelve miRNAs (miR-449a, miR-182, miR-183, miR-187, PC-5p-5933_158, PC-5p-2673_284 and PC-5p-6424_145, PC-5p-20799_45, PC-5p-35677_26, PC-3p-25064_36, PC-3p-82208_8, and PC-5p-108241_6) in the receptive endometrium determined by Solexa deep sequencing or qRT-PCR were higher than those in pre-receptive endometrium (Fig 8). However, Solexa deep sequencing determined that the expression levels of miR-200a in the receptive phase were higher than those in pre-receptive phase, even though the expression levels were nearly equal. What’s more, PC-5p-26648_38 and PC-3p-215602_2 decreased in R library, what were in contrast with the results of Solexa deep sequencing.

## Discussion

### Confirmation and comparison of miRNA data

To date, several miRNAs have been identified in the goat [29, 30], but the information of miRNAs on the development of the receptive endometrium remained quite limited. In the present study, we conducted a detailed overview of experimentally identified miRNAs during the ‘window of implantation,’ which is the receptive phase when the endometrium was permissive to embryo implantation [3, 31]. The analysis resulted in 7,717,603 and 6,307,668 small RNA raw reads obtained from the R (receptive) and P (pre-receptive) endometrium libraries, respectively.

After separating out and discarding junk sequences, we obtained 4,233,874 clean reads representing 284,622 unique sequences from the R library and 5,386,427 clean reads representing 139,040 unique sequences from the P library (Table 1). The length distribution showed that more than 90% of the small RNA sequences were primarily distributed in the 19–24 nt range in both libraries (Fig 1), which was consistent with the typical size of mature mammalian miRNA [32]. These results were also consistent with the typical small RNA distribution in mammals, such as cattle [33], sheep [34] and pigs [35]. The size distribution of the small RNAs from receptive and pre-receptive goat endometrium was similar, but there were differences in the length distribution and abundance between the two libraries. Furthermore, the proportion of total rRNA was used for a quality check of the samples. rRNA proportions are usually less than 60% in high quality plant samples [36] and 40% in high quality animal samples [28]. The proportion of total rRNA in our samples was 7.80% and 3.04% in the R and P libraries, respectively, indicating that the collected endometrium samples were of high quality. The above results suggest that the high-throughput sequencing data were highly enriched for small RNA sequences.

Next, we aligned the clean reads to the miRNAs of all known animals in the miRBase 20.0 database (ftp://mirbase.org/pub/mirbase/CURRENT/). A total of 1,284 miRNAs were identified: 632 of these were co-expressed in both libraries, 1,069 miRNAs were detected in the R library and 847 in the P library. Compared with previous research, more conserved miRNAs (1,069 miRNAs in the R library and 847 in the P library) were identified in this study [28, 37]. There are two principal explanations for these results. The first explanation is that the detected clean reads were aligned to the latest database of miRBase 20.0 released in June 2013 in this manuscript, and 3,355 new hairpin sequences and 5,393 new mature miRNAs were added to the database based on miRBase 19.0 [28]. The second explanation is that the two libraries were compared with all animals in miRBase 20.0 because few goat miRNA sequences are included, and thus the reference data were not species-specific.

### Differential expression of miRNAs in receptive and pre-receptive endometrium of goats

To investigate differences in endometrium activity in the receptive and pre-receptive phases, differentially expressed miRNA were identified in the two constructed libraries. In the present study, 292 miRNAs were up-regulated and 150 miRNAs were down-regulated in the receptive phase compared with the pre-receptive phase (*P*< 0.05), and there were 110 miRNAs increased at least a 2-fold in expression. *Xia* reported that 28 miRNAs were up-regulated at least two-fold and 29 miRNAs were down-regulated at least two-fold during the receptive phase compared with the pre-receptive phase in the rat uterus [12]. Chakrabarty reported that 32 miRNAs were significantly up-regulated at least 1.5-fold and five miRNAs were down-regulated at least 1.5-fold on day 4 of gestation (receptive phase) compared with day 1 of gestation (pre-receptive phase) in the mouse uterus [3]. There are three possible explanations for the increase in differentially expressed miRNAs identified in this study. The first explanation is the differences in species, as the experimental animal in this study was the dairy goat, while previous studies focused on mice and humans [12, 24], whose endometrium structure was different from goat [38]. The second explanation is that the miRNAs in our two libraries were compared with all animals in the miRBase 20.0, which lacks goat miRNAs, so the reference data were not species-specific [28]. Third, in contrast to traditional miRNA identification methods such as direct cloning and computational prediction, Solexa deep sequencing was performed to discover species-specific or poorly expressed miRNAs. This technique has been widely utilized to identify conserved and novel miRNAs in various species [39, 40]. Thus, more potential miRNAs were identified with the development of new sequencing technology.

The miRNA with the largest differential increase in expression was hsa-miR-449a, which had a 113.2-fold increase in the receptive phase compared with the pre-receptive phase. These results suggest that it might play an important role in regulation of endometrium receptivity in goats. Previously, miR-449a had been identified in various types of cancer tissues where it plays a tumor-suppressive role [41], in part through targeting HDAC1 and activating p27 expression [42]. Lize found miR-449 potently induced apoptosis and up-regulated p53 activity [43]. To date, several targets of miR-449a had been identified, such as CDK6, CDC25a, E2F1, CCND1 and BCL2 [44, 45]. Moreover, Paik reported that down-regulation of miR-449a and subsequent up-regulation of CCND1 and BCL2 was a novel mechanism for cell proliferation [46]. miR-449a directly bound to the seed sequence of the LEF-1 (lymphoid enhancer-binding factor-1) 3’ UTR, causing effective repression of its expression and ultimately leading to a subsequent reduction in Sox 9 gene expression [47]. Altogether, these results suggested that miR-449a potentially participated in regulating dynamic changes in goat uterine gene expression patterns that occur during the transition from the pre-receptive to the receptive phase. These result need to be further validated under well-controlled conditions in animal models.

In addition, bta-miR-182 aroused our interest for it was the highest expressed miRNA in receptive endometrium (NE = 3518.63) and increased 14.55-fold compare with pre-receptive endometrium. Studies have identified that miR-182 was significantly up-regulated in endometrial carcinoma tissues (EC) compared with complex atypical hyperplasia, simple hyperplasia and normal endometrial tissues [48–50]. Further study suggested that miRNA-182 binds directly to a conserved 8 bp sequence in the 3’-UTR of its target gene transcription elongation factor A-like 7 (TCEAL7), and then promots cell proliferation by targeting the tumor suppressor gene TCEAL7 and modulating the activity of its downstream effectors c-Myc, cyclin D1 and NFκB in EC cell lines compared with normal endometrial epithelial cells [51]. What’s more, considering that EC was an estrogen-dependent malignancy and that miRNAs were shown to be regulated by estradiol [52], the association between miR-182 and receptive endometrium needs further investigation.

### The specific expressed miRNAs in receptive and pre-receptive goat endometrium

Next, we turned our attention to the specific expressed miRNAs. The miRNA with the highest tissue-specific expression was bta-miR-431_R-3 from the R library. After careful analysis, we found that its sequence was consistent with miR-431, which was initially identified as a central nervous system specific miRNA cloned from the brain tissue of mouse embryos [53]. Wu reported that miR-431 expression induced by nerve injury stimulates regenerative axon growth by silencing Kremen1, an antagonist of Wnt/beta-catenin signaling. Both the gain-of-function of miR-431 expression and knockdown of Kremen1 significantly enhanced axon out growth in murine dorsal root ganglion neuronal cultures [54]. This research suggests that miR-431 might participate in the regulation of endometrial receptivity through the nervous system.

The highly tissue-specific miRNA in the P library was bta-miR-216a_R-2, whose sequence was identical to miR-216a with one mismatch. This miRNA regulates Ybx1 post-transcriptionally, which is a key mechanism for enhanced mRNA translation in kidney cells [55]. Another target of miR-216a was shown to be the tumor suppressor in lung cancer-1 gene (TSLC1) mRNA through three target sites in its 3’ untranslated region [56]. Moreover, miR-216a plays a relevant role in the pathogenesis of cardiovascular disorders and atherosclerosis [57]. Thus, miR-216a potentially regulates the decidualization of the endometrium in the pre-receptive phase, but this hypothesis requires further study.

### Identification of potential novel miRNAs

One of the most important characteristics that distinguishes miRNAs from other endogenous small RNAs is the capacity of the precursor miRNA sequence to fold back into a canonical stem-loop hairpin structure [58]. In this study, a total of 643 miRNA candidates were identified as having the typical miRNA stem-loop secondary structure. Precursor sequences ranged from 50 nt to 123 nt in length and could all be shaped into representative stem-loop structures, with the mature miRNA located at either the 5′ or the 3′end. Additionally, the GC content of pre-miRNA ranged from 23% to 77%, and the free energy (dG) ranged from -80.5 kCal/mol to -17.7 kCal/mol; these factors were considered because unstable pre-miRNA structures were required to produce mature single-strand miRNAs [59]. There were 33 novel miRNA candidates with lengths ranging from 18 nt to 24 nt, with the majority at 22 nt. The numbers of these miRNA were shown to be greater than 10. Other miRNAs were not considered for this study because of their low abundance in the goat endometrium.

### GO enrichment and KEGG pathway analyses

Previous studies showed that miRNAs modulated gene expression by inhibiting mRNA translation or regulating mRNA degradation at the posttranscriptional level based on Watson-Crick pairing between the 5’ end of the miRNA (2–8 nt, the “seed” region) and the 3’ un-translated regions (3’ UTR) of target mRNAs [60]. An animal miRNA was predicted to target at least 200 genes on average because of the short length of the seed region [61, 62]. However, 7,608 annotated mRNA transcripts were predicted as putative target genes for the 143 differentially expressed miRNAs in the present study, suggesting a miRNA targeted 53 genes on average. The small number of predicted target genes may be due to a lack of relevant data on goat genes compared with other animals (i.e., human and rat).

GO is an international standardized classification system for gene function, supplying a set of controlled vocabulary to comprehensively describe the properties of genes and gene products [28]. GO analysis provides insight into the molecular functions of genes in various biological processes [63]. In the present study, GO enrichment analysis revealed that 58.10% of the genes were involved in biological processes, 13.64% of the genes were annotated to intracellular component ontology, and 28.26% of the genes referred to molecular functions. Moreover, the miRNA-gene network further revealed core genes involved in remodeling of the endometrium, such as induction of apoptosis by extracellular signals, cell cycle checkpoint, vesicle-mediated transport, intracellular protein transport and maternal processes involved in female pregnancy.

In organisms, genes usually interact with each other to play different roles in certain biological functions [28]. KEGG pathway analysis could facilitate the understanding of the biological functions of genes [64, 65]. A critical event during embryo implantation is the extensive tissue remodeling at the maternal-fetal interface, which was characterized by cell proliferation, cell migration and cell adhesion [1, 66]. In the present study, ubiquitin mediated proteolysis, natural killer cell mediated cytotoxicity, T cell receptor signaling pathway, Fc gamma R-mediated phagocytosis, and apoptosis were involved in the significantly enriched miRNA-associated pathways in receptive goat endometriums. These significantly enriched pathways might imply that the organism was coping with the criteria of the endometrium phase during the ‘window of implantation’ in goats. In summary, GO and KEGG analyses provide a better understanding of the cellular components, molecular functions and biological processes of target genes [30], and provided a reference for future research

### Validation of miRNA expression with Stem-loop RT-qPCR

We performed stem-loop qRT-PCR, which is a reliable method used to detect, measure and validate the expression levels of miRNAs. The expression trends of 8 miRNAs measured by Solexa deep sequencing were consistent with those determined by stem-loop qRT-PCR, but the differences in expression levels between the two methods did not agree well. Moreover, the expression patterns of miR-200a measured by Solexa deep sequencing were opposed to those determined by stem-loop RT-qPCR.

This inconsistency might result from differences in the two methods. The expression levels of miRNAs measured by Solexa deep sequencing required the following seven steps: RNA extraction, small RNA fractionation, 5’ adaptor ligation, 3’ adaptor ligation, RT-PCR, sequencing, and data analysis; thus, the information from some sequences might be lost or produce low-quality tags that were filtered during data analysis [58]. Moreover, some miRNAs might be hard to sequence because of their physical properties [67]. Stem-loop RT-qPCR was a new method designed to detect and quantify mature miRNAs in a fast, specific, accurate, and reliable manner [58]. It was designed to quantify miRNAs using three steps: RNA extraction, Stem-loop RT (reverse transcription) and RT-qPCR. Moreover, the stem-loop RT primer contained a highly stable stem-loop structure that lengthened the target miRNA, and the RT product was amplified and monitored in real time by use of a specific forward primer and a universal reverse primer [68]. Thus, Solexa deep sequencing was considered inferior to RT-qPCR in terms of miRNA quantification [58]. Nevertheless, the trends of expression of 12 miRNAs detected by the two methods in our study were consistent, suggesting that the majority of the miRNA expression data could represent actual miRNA expression levels.

## Conclusion

We obtained high-quality miRNA expression profiles from pre-receptive endometrium and receptive endometrium, and predicted 545 known and 33 novel miRNAs in endometrium of dairy goats for the first time. Target gene predictions for the 143 differentially expressed miRNAs, functional annotations and pathway analyses in GO and KEGG databases could contribute to a better understanding of the miRNA mediated regulation of target genes in the development of the endometrium receptivity.

## Materials and Methods

### Ethics statement

All animals in this study were maintained according to the No. 5 proclamation of the Ministry of Agriculture, P. R. China. And animal protocols were approved by the Review Committee for the Use of Animal Subjects of Northwest A&F University.

### Study design and tissue collection

A total of 20 healthy 24-month-old multiparous dairy goats (Xinong Saanen) were induced to estrous synchronization for this study. The first day of mating was considered to be Day 0 of pregnancy. Gestational days 5 and 15 were important time points for the embryo implantation in goats [38]. The experimental goats were observed 3 times daily to ascertained estrous sign and mated naturally twice in estrus. And then the goats were euthanized when the goats lost consciousness caused by intravenous injection of barbiturate (30mg/kg) at gestational day 5 (pre-receptive endometrium) and gestational day 15 (receptive endometrium). Endometrium samples from 10 goats at gestational day 5 and 10 goats at gestational day 15 were obtained from the anterior wall of the uterine cavity. All tissue samples were washed briefly with PBS (Phosphate Buffered Saline) and then immediately frozen in liquid nitrogen.

### Small RNA library construction and sequencing

Total RNA was extracted from every sample using Trizol reagent (Invitrogen, CA, USA) following the manufacturer’s procedure. The total RNA quantity and purity were analysis of Bioanalyzer 2100 (Agilent, CA, USA) and RNA 6000 Nano LabChip Kit (Agilent, CA, USA) with RIN number> 7.0. Approximately 1 ug of total RNA were used to prepare small RNA library according to protocol of TruSeq Small RNA Sample Prep Kits (Illumina, San Diego, USA). The total RNA with lowest quality was not used for further study from the pre-receptive endometrium samples and receptive endometrium samples, respectively. P library was constructed by mixing the nine samples into three samples with the same concentration, performing the single-end sequencing (36 bp) on an Illumina Hiseq2500 (Illumina, San Diego, USA) thrice at the LC-BIO (Hangzhou, China) following the vendor’s recommended protocol, and R library was constructed in the same way.

Data processing followed the procedures described in a previous study [69] by *LC Sciences Service*. Briefly, the raw reads were subjected to the Illumina pipeline filter (Solexa 0.3), and then the dataset was further processed with an in-house program (ACGT101-miR, LC Sciences, Houston, Texas, USA) to remove adapter dimers, junk, low complexity, common RNA families (rRNA, tRNA, snRNA and snoRNA) and repeats.

### Identification of known miRNAs

Unique sequences 18–26 nt in length were mapped to specific species precursors in miRBase 20.0 (ftp://mirbase.org/pub/mirbase/CURRENT/) by BLAST search to identify known miRNAs and novel 3p’ and 5p’ derived miRNAs. The remaining sequences were mapped to other selected species precursors (with the exclusion of specific species) in miRBase 20.0 by BLAST search, and the mapped pre-miRNAs were further BLASTed against the specific species genomes to determine their genomic locations. The above two were defined as known miRNAs. On the basis of analysis for detected miRNAs, we further analyzed the conservatism of these miRNAs with selected species on frequency for the number in statistics.

### Identification of potential novel miRNAs

Sequencing reads that did not match any known miRNAs were further analyzed to discover novel miRNAs using the characteristic hairpin structure of the miRNA precursor [28, 58]. To determine whether these unmapped small RNA reads were genuine miRNA, the unmapped sequences were BLASTed against the specific genomes, and the sequences containing hairpin RNA structures were predicated from the flanking 80 nt sequences using RNA fold software (http://rna.tbi.univie.ac.at/cgi-bin/RNAfold.cgi/). The criteria for secondary structure prediction were described by Mi and Lian [27, 70].

### Analysis of differential expressed miRNAs

To compare differentially expressed miRNAs in the receptive endometrium (R library) and pre-receptive endometrium (P library) of goats, the expression abundances of miRNAs in the two samples were normalized to obtain the expression of transcripts per 1,000,000. And the formulae was: Normalized expression (NE) = (Actual miRNA count/Total count of clean reads) × 1,000,000.

When the NE of a certain miRNA was 0 in one of the two libraries, we revised the 0 to 0.001 for the comparative, and the miRNA whose NE was less than 1 in both libraries were discarded described in detail previously [28]. The differential expression of miRNA based on normalized counts was analyzed using Fisher’s exact test, and the significance threshold was set to be 0.05 for each test. The fold-change and *P*-value for each miRNA were calculated based on the normalized expression using the formulae as described previously [28] and shown below:

Fold change = Log2 (R−NE/P−NE).

*P*-value formula:

$$P\left(x|y\right)=\left(\frac{N_{2}}{N_{1}}\right)\frac{\left(x+y\right)}{x!y!{\left(1+\frac{N_{2}}{N_{1}}\right)}^{\left(x+y+1\right)}}\overset{C\left(y\leq y_{\text{min}}|x\right)=\sum_{y=0}^{y\leq y_{\text{min}}}p\left(y|x\right)}{\underset{D\left(y\geq y_{\text{min}}|x\right)=\sum_{y\geq y_{\text{max}}}^{\infty }p\left(y|x\right)}{}}$$

N1 and X represent the total number of clean reads and normalized expression level of a given miRNA in the small RNA library generated from P library, respectively, and N2 and Y represent the total number of clean reads and normalized expression level of a given miRNA in the small RNA library generated from the R library, respectively.

### Target genes prediction of differential expression miRNAs

Two computational target prediction algorithms (TargetScan 5.0 c http://www.targetscan.org/cgi-bin/targetscan/data.download.cgi?db=vert.61 and miRanda 3.3a c http://www.microrna.org/microrna/home. do) were used to predict target genes of differentially expressed miRNAs. Only when the target was identified by both programs, it was considered to be the predicted target for a given miRNA.

### GO enrichment and KEGG pathway analysis of target genes

GO enrichment analysis was performed on predicted target gene candidates of differentially expressed miRNA. This method mapped all target gene candidates to GO terms in the database (ftp://ftp.ncbi.nih.gov/gene/DATA/gene2go.gz), calculated gene numbers for each term and used hyper-geometric tests to find significantly enriched GO terms in target gene candidates. The results were compared with the reference gene background and to genes corresponding to certain biological functions.

The calculating formula:

$$P=1-\overset{m-1}{\underset{i=0}{\sum }}\frac{\left(\frac{M}{i}\right)\left(\frac{N-M}{n-i}\right)}{\frac{N}{n}}$$

The N represented the number of GO annotated genes in genome, *n* represented the number of differentially expressed genes in N. M represented the number of particular GO annotated genes in genome, *m* represented the number of particular GO annotated genes expressed differentially in M [30]. And then P was less than 0.05 was used as the threshold to judge significant enrichment GO term in this study.

KEGG pathway analysis facilitates the understanding of biological functions of genes [28]. The analysis revealed the main pathways in which the target gene candidates were involved. KEGG pathway analysis (http://www.genome.jp/kegg/) used the same formula as GO analysis to determine target gene candidates. Here, N was the number of all genes with KEGG annotation, *n* was the number of target gene candidates in N, M was the number of all genes annotated in a certain pathway and *m* was the number of target gene candidates in M [30].

The miRNA-gene network was constructed using Cytoscape Software [71] (Cytoscape_v2.8.1, http://www.cytoscape.org/) to analyze the interactions between miRNA and genes. Firstly, the relationship and the interactions between miRNAs and genes were determined by using their differential expression values in the databases of TargetScan 5.0 and miRanda 3.3a. And then we used the random variance model (RVM) corrected t-test (*P*< 0.05) to identify the differently expressed genes and miRNAs from the gene expression profiles. The overlap between the genes whose expression was induced and the target genes whose expression was reduced by miRNA were then chosen to construct the network based on the two respective expression values by using the miRNA–mRNA modules via population-based probabilistic learning methods [72, 73]. A = [ai, j] was defined as the adjacency matrix of miRNA and genes based on the attribute relationships between target genes and miRNA, represented the relationship weight between gene i and miRNA j [74].

### Validation of differential expression miRNAs using Stem-loop RT-qPCR

Stem-loop qPCR was used to validate the known and potential novel miRNAs in present manuscript. Total RNA was extracted using Trizol reagent (TaKaRa, Dalian, China) following the manufacturer’s instructions. Total RNA was converted to cDNA with a Stem-loop primer using the Prime Script RT reagent Kit with gDNA Eraser (TaKaRa, Dalian, China) according to the manufacturer's instructions. The reverse transcription reaction system were: added 5 μl total RNA (800 ng), 2 μl 5× gDNA Eraser Buffer, 1μl gDNA Eraser and RNase-Free dH_2_O to a final volume of 10 ul, incubated the mixture at 65°C for 3 min, and then added 4μl 5× Prime Script Buffer 2 (for real-time PCR), 1μl PrimeScript RT Enzyme Mix 1, 1μl Stem-loop primer and 4 μl RNase-Free dH_2_O to a final volume of 20 ul, incubated the mixture at 42°C for 15 min, followed by 85°C for 5 sec. The cDNA products were diluted 1:10 (v/v) with sterile water.

RT-qPCR was performed using the Bio-Rad CFX 96 Real Time Detection System and SYBR Green PCR Master Mix (TaKaRa, Dalian, China) in a 20 μl reaction according to the manufacturer’s instruction. The PCR mixture included 4 ul cDNA for each miRNA, 2 μl primer mix (10 uM), 10 μl 2× SYBR Green Mix with ROX, and 4 μl RNase-Free dH_2_O. The reaction mixtures were amplified in a 96-well plate at 95°C for 10 min, followed by 40 cycles of 95°C for 10 s, 60°C for 1 min, and all reactions were performed in triplicate. 18S rRNA was used as the reference, and all primers for the Stem-loop RT-qPCR are shown in S7 Table. The relative expression levels of the miRNAs were calculated using the equation N = 2^-ΔΔCt,^ ΔΔ = (CT _miRNA_ − CT _18s_) _R_ − (CT _miRNA_ − CT _18s_) _P_. The Ct (cycle threshold) was defined as the cycle number at which the fluorescence intensity passed a predetermined threshold [75].

### Statistical analysis

All the data were processed with SPSS 17.0 (SPSS Inc., Chicago, IL, USA). One-way ANOVA was used to compare the differences, and the method of the least significant difference (LSD) was used for further analysis, and the differences were considered significant when *P* was <0.05 and very significant when *P* was <0.01.

## Supporting Information

S1 Table Summary of known miRNA in this study. (XLS)

S2 Table Conservation of the identified miRNA with other species. (XLS)

S3 Table Summary of potential novel miRNA in this study. (XLS)

S4 Table Results of predicted targets of differently expressed miRNAs. (XLS)

S5 Table Targets enrichment analysis of GO. (XLS)

S6 Table KEGG significant analysis. (XLS)

S7 Table Stem-loop RT-PCR Primer in this study. (XLS)
